# Effects of wildfire disaster exposure on male birth weight in an Australian population

**DOI:** 10.1093/emph/eov027

**Published:** 2015-12-30

**Authors:** M. H. O’Donnell, A. M. Behie

**Affiliations:** ^1^School of Archaeology and Anthropology, College of Arts and Social Sciences, the Australian National University, Canberra 0200, Australia

**Keywords:** disaster, birth weight, macrosomia, maternal stress, gestational diabetes

## Abstract

**Background and objectives***:* Maternal stress can depress birth weight and gestational age, with potential health effects. A growing number of studies examine the effect of maternal stress caused by environmental disasters on birth outcomes. These changes may indicate an adaptive response. In this study, we examine the effects of maternal exposure to wildfire on birth weight and gestational age, hypothesising that maternal stress will negatively influence these measures.

**Methodology***:* Using data from the Australian Capital Territory, we employed Analysis of Variance to examine the influence of the 2003 Canberra wildfires on the weight of babies born to mothers resident in fire-affected regions, while considering the role of other factors.

**Results***:* We found that male infants born in the most severely fire-affected area had significantly higher average birth weights than their less exposed peers and were also heavier than males born in the same areas in non-fire years. Higher average weights were attributable to an increase in the number of macrosomic infants. There was no significant effect on the weight of female infants or on gestational age for either sex.

**Conclusions and implications***:* Our findings indicate heightened environmental responsivity in the male cohort. We find that elevated maternal stress acted to accelerate the growth of male fetuses, potentially through an elevation of maternal blood glucose levels. Like previous studies, our work finds effects of disaster exposure and suggests that fetal growth patterns respond to maternal signals. However, the direction of the change in birth weight is opposite to that of many earlier studies.

## BACKGROUND AND OBJECTIVES

A growing body of work considers the effects of maternal exposure to stressors on fetal health, principally in terms of birth weight and gestation length, with an increased incidence of both low birth weight (birth at <2500 g) and prematurity (birth at <37 weeks gestation) being documented in populations exposed to major stressors during gestation [1–17]. Preterm birth and low birth weight are substantial public health issues that have negative impacts on infant survival [18]. Fetal exposure to maternal stress also has deleterious effects on fetal development, most likely through the programming of downstream effects, including increased baby stress response [19–26], decreased baby immune response [27], childhood obesity [28] and poorer developmental outcomes during childhood [17, 29–31].

Maternal stress is likely linked to lower weights and shorter gestations because the physiological stress response creates an energetic cost that adds to the metabolic load of gestation. An evolutionary perspective suggests that this greater load encourages reduced maternal metabolic investment in the fetus in order to aid the mother’s own chance of survival and thus her future reproductive potential [32]. Theoretically, this reduced investment results in lower birth weights and shorter gestations.

Consistent with this evolutionary theory, natural experiments demonstrate that maternal exposure to stress is associated with reduced birth weights and gestational age [33]. For example, in studies of those mothers affected by the 1998 Quebec Ice Storm, Auger *et al.* [34] and Dancause *et al.* [4] find decreases in gestational length and birth weights following storm exposure. Similarly, Xiong *et al.* [14] find increases in the risk of preterm birth and low birth weight neonates in a New Orleans population who were highly exposed to Hurricane Katrina in 2005. A similar result was found after Hurricane Andrew in 1992 in southern Florida where an increased risk of preterm birth was also found [35].

Likewise, Oyarzo *et al.* [36] find an increased risk of low birth weight among neonates of women exposed to a 2010 Chilean earthquake, supporting the earlier findings of Torche and Kleinhaus [13] who find an increase in preterm birth among girl children following a 2005 Chilean earthquake. In our previous examination of the 2009 Black Saturday fire in Victoria, Australia, we also find an increase in preterm births and low birth weight infants among children born in the immediate aftermath of the fire [37]. Similarly, research in mother-fetus dyads who experience war or terrorism also finds consistent patterns of lower birth weights in exposed cohorts [8, 38].

Notably, there are some exceptions to the overall trend of decreases in birth weight and gestational age. In addition to the trend of lighter and earlier neonates following the 2010 earthquake in Chile, increases in the incidence of macrosomic infants were also found and were attributed to underlying gestational diabetes mellitus (GDM) and maternal obesity [36]. Similarly, our research on the Black Saturday fires also found a secondary pattern of macrosomic and post-term births during the fire aftermath, potentially reflecting changes in health care decisions [37]. Further, analysis of Californian births following the 11 September 2001 terrorist attacks in New York shows that fetuses exposed later in gestation also had an increased likelihood of post-term birth, identified in that study as a potential adaptation [39].

From an evolutionary perspective, it is also understood that stress-related differentials in maternal investment exist depending on the sex of offspring [40]. Sex differences in optimum maternal investment levels become more apparent under stochastic conditions, with male fetuses appearing more sensitive to changes in the uterine environment [41] and, accordingly, more prone to early fetal loss when environmental quality declines [42]. This pattern is demonstrated in studies which examine environmental disasters. For example, decreases in secondary sex ratio, indicative of increased male fetal loss, are documented following earthquakes [13, 43, 44], extreme heat and cold [45] and terrorist attacks [46, 47]. This pattern of loss is consistent with both theories of increased male genetic frailty and patterns of greater male responsivity to environmental stress *in utero*, as well as in early life [48].

In this study, we examine patterns in average birth weights and gestational ages following the 2003 Canberra Wildfires in the Australian Capital Territory (ACT). Wildfires expose populations to both physiological and psychosocial stressors. Some such stressors include experiences of extreme heat and poor air quality, both of which have independent effects on pregnancy [49, 50]. Psychosocial stressors of fear, threat and loss are also major factors and these may persist for months or years after the fire event impacting pregnancy outcomes.

## METHODOLOGY

### Event description

On 18 January 2003, the Canberra fires burnt part of the southern and western suburbs of Canberra, Australia’s national capital, located in the ACT. The Canberra fires were the northern most tip of an 800 km fire line known collectively as the Australian Alps fires. The Canberra fires killed four people, injured 489 people, destroyed ∼500 homes and triggered the evacuation of 5000 residents. A further 50 000 residents lost essential services, representing around 15% of Canberra’s total population [51].

### Data collection

Following ethics approval from The Australian National University Human Research Ethics Committee and the ACT Government Human Research Ethics Committee’s Low Risk Sub-Committee, and approval of data access by the ACT Chief Health Officer, the study used unit record data managed by the ACT Government Health Directorate Epidemiology Section (Population Health Informatics) to examine birth weights and gestational ages in the ACT between 2000 and 2010.

Clinical birth records in Australia are collected and administered for almost all births by hospitals and midwives [52]. Birth record data is collected by hospital staff, midwives or medical staff from maternal self-report, clinical and procedural documentation. Anthropometrics are collected by trained personnel using appropriate equipment. Gestational age is a clinical estimate based on maternal recall of last menstrual period, ultrasound imaging or a combination of both. Measures of maternal factors, such as cigarette smoking, alcohol consumption, marital status and Aboriginal or Torres Strait Islander status, are self-reported and, as such, may contain inaccuracies. Maternal age is derived from health records. Previous pregnancy is self-reported and verified by cross-referencing with health records. In total, 48 408 births were represented in the ACT birth records from 2000 to 2010. Date of birth was only known by the month and year of birth, thus an estimated trimester of exposure is used in this analysis.

### Analysis

Indigenous status was recoded into a binary variable as either ‘Indigenous’ (including women who identify as Aboriginal and/or Torres Strait Islander) or ‘not Indigenous’. Similarly, plurality was recoded as either ‘singleton’ or ‘multiple’, with the latter category including all multiples. Lastly, divorced, separated and widowed mothers were combined in the analysis of the influence of marital status, due to small cell sizes. ‘Married’ mothers included those in common law or same-sex relationships. Maternal smoking in all analysis refers to reported smoking at any point during gestation.

Three levels of fire exposure were defined using the mothers’ reported Statistical Local Area of residence at the time of birth. Mothers’ reported location of residence was used as a proxy measure of disaster exposure. The three levels of exposure we used were: ‘severely affected’, which included mothers who were resident in areas where both deaths and property damage occurred; ‘moderately affected’, which included mothers from areas where property damage (only) occurred and ‘least affected’, which was the remainder of the residential areas of Canberra, where no fire damage occurred.

Because disaster impacts can be difficult to define geographically and people outside the affected zones may also experience substantial stress, the analysis included inter-year comparisons of the same areas as well as intra-year comparison between levels of disaster exposure. This allows births in each region to be compared to those in the same region in pre- and post-fire years.

Analysis of variance (ANOVA) was used to examine the influence of residential location on birth weight and gestational age in the year of the fire (2003), with the three previous years (2000–02) and seven following years (2004–10), while also considering the influence of maternal age, maternal smoking during gestation, maternal Indigenous status, maternal marital status, baby’s plurality, and maternal previous pregnancy status (i.e. primigravida versus multigravida). Research indicates that multiple births, older maternal age, positive maternal Indigenous status, maternal smoking and single marital status have a depressive effect on birth weight [53, 54]. Second and third children tend to report higher birth weights, although this effect is modified by the birth weight of the first born child and can invert as the number of subsequent children rises.

Births in 2002, 2003 and 2004 were divided into quarters to allow the effects of timing of exposure to be examined more closely, using approximate trimesters of exposure. Because previous studies indicate some sex differences in stress responsivity [13, 55] this analysis was conducted separately for each sex for every year in the sample (years 2000–2010), as well as over the full eleven year period using an interaction term between maternal residential location and the timing of birth. Where ANOVAs indicated the presence of a significant effect, we then used chi-square tests of independence.

## RESULTS

Table 1 shows descriptive statistics for the sample population in 2003, the fire year.

**Table 1.** Descriptive statistics for the two dependent variables (gestational age and birth weight) and independent variables included in this analysis for 2003 (the fire year) divided by degree of estimated fire exposure

Variable	Total (*n* = 4107)		Heavily affected (*n* = 255)		Moderately affected (*n* = 2583)		Least affected (*n* = 1269)		*P*-values arising from ANOVA
***Continuous variables***	**Mean**	**SD**	**Mean**	**SD**	**Mean**	**SD**	**Mean**	**SD**	
**Birth weight (grams)**	3408	620.3	3549	616.2	3394	634.2	3409	588.5	<0.001
**Gestational age (weeks)**	39.03	2.33	39.28	1.763	38.97	2.501	39.1	2.049	0.68
Maternal age (years)*	30.23	5.28	30.96	4.852	29.82	5.299	30.92	5.24	<0.001

***Categorical variables (expressed as percentages)***									***P*-values arising from chi-square**

Mothers in a couple*	90.75		92.16		90.44		91.10		<0.001
Indigenous mothers*	1.42		0.39		1.48		1.51		0.006
Multiple births*	2.73		0.78		3.14		2.29		0.10
Mothers smoked in gestation*	12.02		9.02		13.79		9		<0.001
Primigravida mothers*	35.11		30.20		34.49		37.35		<0.001
**GDM prevalence#**	4.39		3.53		4.61		4.26		0.26

The right-hand column shows the results of tests for differences between levels of fire exposure for each variable. Variables marked with an asterisk were included in the ANOVA analyses and those marked with a hash were considered in subsequent chi-square analyses.

### Birth weight analysis

ANOVAs for each year showed that average male birth weights were 197 g heavier in the severely affected area in the year of the fire (2003) (F = 5.73, *P *< 0.003, df = 2), 18 g heavier in the severely affected area in 2005 (F = 3.06, *P *= 0.05, df = 2), 47 g heavier in the severely affected area in 2008 (F = 3.19, *P *= 0.04, df = 2) and 56 g lighter in the severely affected area in 2010 (F = 3.52, *P *= 0.03, df = 2) when compared to those reported in the moderately affected area.

ANOVAs for each year also showed that average female birth weights were 163 g greater in the severely affected area in 2008 (F = 5.71, *P *< 0.003, df = 2), 117 g greater in the severely affected area in 2009 (F = 3.47, *P = 0*.03, df = 2) and 160 g greater in the severely affected area in 2010 (F = 5.66, *P *< 0.004, df = 2) when compared to those reported in the moderately affected area.

**Figure 1.**
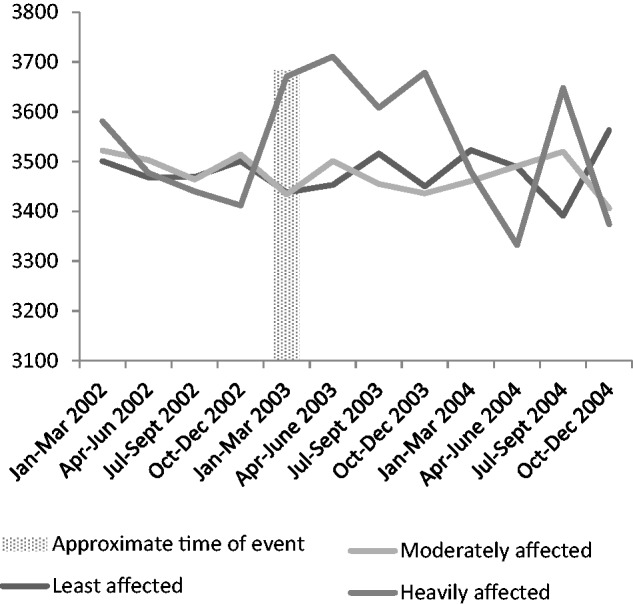
Means for birth weight arising from ANOVA analysis of males (only) at three levels of estimated fire exposure between 2002 and 2004, with the fire and fire aftermath (2003) shown by the shaded area. Babies born between the fire (18 January) and March 2003 are estimated to be exposed in the third trimester, those born between April 2003 and June 2003 as exposed in the second trimester and those born between July 2003 and September 2003 as exposed in the first trimester. Babies born between October 2003 and December 2003 would have been conceived in the fire aftermath

The analysis of males born in 2003 (the year of the fire) showed that being in the severely affected area in the year of the fire was only significant for male offspring (F = 5.73, *P *= 0.003, df = 2). A further ANOVA which included data from all years indicates that exposed male neonates were heavier throughout 2003 than males born to mothers residing in the same areas in the previous and following years (F-value = 1.58, *P *= 0.01, df = 2), as well as being heavier than males born elsewhere during 2003 (Fig. 1). Males born to mothers who resided in severely affected areas had a predicted mean birth weight of 3657 g, compared with 3460 g for those born to mothers in affected areas and 3454 g for those born to mothers in unaffected areas.

Other significant factors detected in the 2003 ANOVA on birth weight were consistent with the literature: those mothers who smoked during gestation (F = 49.75, *P *< 0.001, df = 1) or reported as single mothers (F = 7.47, *P *= 0.006, df = 1) had babies with lighter mean birth weights [53]. Also consistent with the literature, multiples were significantly lighter than their singleton peers (F = 153.68, *P *< 0.001, df = 1) and primigravida mothers had lighter offspring than multigravida mothers (F = 14.97, *P* < 0.001, df = 1) [53]. Older maternal age increased average predicted mean birth weight (F-value = 6.3, *P* = 0.01).

**Figure 2.**
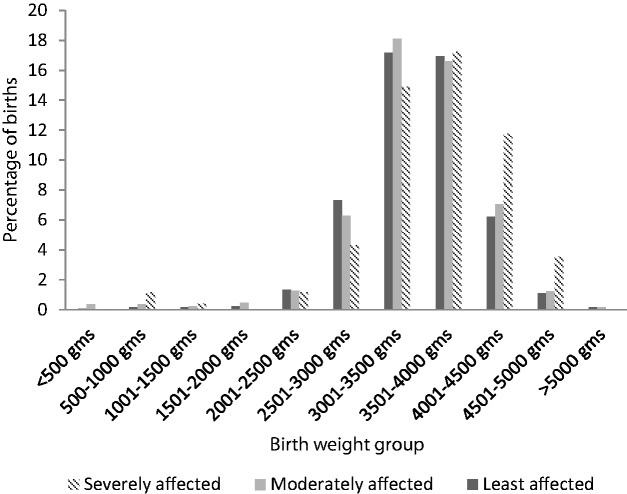
Plot of the percentage of births in each weight category by fire exposure in 2003, a chi-square test indicates that these differences are significant at *P = *0.02. In the severely affected area, there were increases in birth weights between 4000 and 5000 g, which were not offset by the increases in birth weights between 500 and 1500 g

A chi-square test found that the increase in average birth weight was associated with a significant increase in the number of male neonates born at >4000 g (permutation corrected *P *= 0.02, uncorrected *P* = 0.01) with the largest difference occurring in individuals born between 4501 and 5000 g (shown in Fig. 2, below). There was also an increase in exposed male infants born at between 500 and 1500 g that was insufficient to offset the overall increase in average weights.

### Gestational age

We found no significant influence of being present in a fire affected area during 2003 on average gestational age. ANOVAs indicated that there was no significant differences in gestational age between mothers of male or female infants who lived in differently affected geographical areas in the year of the fire.

Although we observe an increase in gestational age when the birth weights increases, this correlation is not statistically significant for those babies born between January and March 2003 (i.e. those born immediately after the fire and mostly exposed in the third or late second trimester) and between October and December 2003 (i.e. those exposed very early in the first trimester or conceived in the fire aftermath). This positive relationship was not seen in neonates born between April and September 2003 (i.e. those exposed in the first and second trimesters). Accordingly, the relationship between birth weight and gestational length appears disrupted. While infants who were in the third or late second trimester at the time of the fire show a consistent relationship between gestational length and birth weight, the strength of this relationship declines over the remainder of 2003 (i.e. children exposed earlier in gestation or conceived in the fire aftermath) before becoming more consistent again from early 2004 onwards.

### Maternal health

Because of the unusual pattern of higher birth weight, we explored the presence of other maternal conditions with a known relationship to birth weight disruptions including gestational diabetes and maternal smoking. Chi-square tests found no significant differences in the prevalence of GDM between the three defined areas of fire exposure in 2003 among mothers of male neonates (*P* = 0.81). Similarly, there were no significant differences in the prevalence of pre-existing maternal diabetes mellitus across the three levels of fire exposure (*P* = 0.99).

Although maternal smoking was controlled for in the analysis, unreported smoking was considered as a possible relevant factor. Maternal smoking declined sharply in the ACT during the first 6 months of 2003 (*P *< 0.001), but these declines were concentrated outside the severely affected area.

## DISCUSSION

Our analysis indicates that being present in the severely impacted area during gestation (as indicated by reporting a residential address in the severely affected area at birth) had a significant effect on the birth weights of exposed male fetuses, resulting in a pattern of higher birth weights with only a partially corresponding and non-significant increase in gestational length, largely in those fetuses exposed in the later stages of gestation. Because birth dates were only known by month and year, it is not possible to be more specific on the effects of exposure timing. The absence of a significant increase in gestational length suggests accelerated *in utero* growth. Although there was also a pattern of higher and lower birth weights in the severely affected area in other years, the smaller magnitude of these changes, in addition to their greater variation in direction, sex differentiation and temporal dislocation suggests these changes are not fire related and, indeed, may not have a common cause.

While we accounted for the effects of reported maternal smoking, this analysis cannot include unreported maternal smoking. During 2003, there was a decline in reported maternal smoking but this was concentrated outside the severely affected area. Assuming that unreported smoking follows similar patterns to reported smoking, changes in actual maternal smoking are unlikely to explain the increase in birth weights observed and nor would maternal smoking necessarily provide an explanation for the sex differentiation of the effect.

Because macrosomia poses important health risks to both mother and neonate, potential causes of macrosomia deserve close attention. In the short term, macrosomia is associated with higher birth risks, including unplanned caesareans, assisted delivery, shoulder dystocia, newborn asphyxia and perineum tears [56]. Over the longer term, macrosomic infants report higher rates of obesity and metabolic disorders, indicating fetal programming effects [56, 57].

Three studies have previously reported secondary patterns of higher birth weights or lengthened gestation following disasters: Oyarzo *et al.* [36] after a 2010 Chilean earthquake, ourselves after the 2009 Black Saturday wildfires in Australia [37], and Margerison-Zilko *et al.* [39] following the September 11 terrorist attacks. In our earlier study, we hypothesized that patterns of longer gestation might reflect altered maternal decision making regarding obstetric intervention following disasters [37]. Disasters are understood to reduce individuals’ sense of control and one disaster study reports that women feel their babies are safer *in utero* [58]. Such psychosocial effects might lead to women delaying intervention and thus prolonging gestation. Similarly, Margerison-Zilko *et al.* [39] suggest that prolonged gestation is relevant, in this case as a protective adaptation to a stochastic environment. However, in the case of the Canberra fires, lengthened gestation was only partially correlated with birth weight changes and alterations in maternal decision-making would not explain the sex differentiation found, making maternal decisions an insufficient explanation in this case. Any protective adaptation that prolongs gestation would also potentially incur survival risks associated with prolonged gestation, macrosomia and late parturition. Oyarzo *et al.* [36] suggest that the increases in macrosomia found in their study reflect the underlying prevalence of GDM and obesity in the study population. Our current analysis shows that the prevalence of GDM was not different between women in the three levels of fire exposure, with no increased prevalence of GDM among the mothers of male neonates between the three levels of fire exposure.

The presence of macrosomia indicates a dysregulation of maternal glucose levels. While we excluded diagnosed GDM through our analysis, it is important to note that GDM is usually screened for only once during gestation (at around 25–26 weeks) in otherwise low-risk pregnancies and has few obvious symptoms. Although high birth weights were recorded both in babies exposed to the fire before screening and in babies exposed after screening, the absence of a GDM diagnosis does not necessarily indicate the absence of any blood glucose elevation. Maternal glucose and fetal weight correlate in non-diabetic mothers, indicating that persistent increases in maternal blood glucose can increase fetal weight, even when blood glucose levels remain below those required for GDM diagnosis [59, 60]. We suggest that an elevation in maternal blood glucose among exposed women may have occurred though stress-related changes to glycogenesis or glucose metabolism.

Normal pregnancy triggers the action of placental steroid hormones, including corticotropin-releasing hormone [61] and placental adrenocorticotropic hormone (ACTH) [62]. Although a placental product, placental ACTH remains bioactive in the maternal bloodstream [62] and thus functions to raise maternal blood glucose as a normal feature of pregnancy. However, stress increases maternal cortisol independently of these systemic changes in maternal endocrinology. There is evidence of a modest relationship between maternal self-report of anxiety or stress and cortisol in pregnant women [22, 62–64]. Although mothers’ experience compensatory dampening in cortisol responsivity as pregnancy progresses [17], the timing of clinically significant reductions in responsivity remains unclear and some degree of responsivity persists throughout pregnancy [22].

Stress-related increases in cortisol levels would be predicted to increase circulating blood glucose via accelerated gluconeogenesis or through the disruption of glucose metabolism [65]. As such, maternal stress can result in heightened blood glucose levels beyond the increases that are usual in pregnancy. As maternal glucose is able to traverse the placental barrier, a more glycaemic intrauterine environment could result, increasing macrosomia risk.

Also potentially disrupting glucose metabolism are changes in food intake and physical activity. Both increased food intake and increased consumption of low-nutrition but high-calorie foods have been documented as a behavioral response to stress during pregnancy [66, 67]. Increased maternal food consumption was significant only in energy-dense food categories, such as bread, sweets, oils and fats [67]. This study was unable to account for the diets of participants; however, it is likely that similar behaviors may have occurred in the disaster-exposed women, particularly as the fire did not interrupt food supplies.

Although little is known about physical activity patterns of pregnant women during wildfires, official advice to stay indoors (due to smoke and heat) or to relocate to an evacuation centre would both have the effect of reducing normal physical activity. If reductions in physical activity occurred these would also likely alter glucose metabolism.

Maternal body weight, and weight gain in pregnancy, are also good predictors of macrosomia [59]. We were unable to account directly for maternal body weight, or other socio-economic factors that might be expected to influence maternal wellbeing. However, our analysis compares the fire-exposed cohort with birth cohorts in the same areas in the preceding and following years. Because these cohorts include both some of the same women and women who experience broadly consistent socio-economic circumstances, the lack of any persistent pre-existing regional differences in birth weights reinforces the likely presence of a fire effect.

The Hypothalamus–Pituitary–Ovarian axis can act to upregulate steroid hormone production when nutritional energy is abundant [61]. This indicates a behavioral link between the stress response, appetite and resulting food consumption. Under conditions of high energy availability, over-macronutrition combined with the actions of stress hormones may have created a higher than usual supply of glucose to the fetus.

It is well documented that male fetuses are more responsive to maternal stress than female fetuses, often measured through rates of early pregnancy loss [42, 68] or changes to birth weight [48] associated with maternal stress. In terms of birth weight, these changes have tended towards males exhibiting greater lowering of birth weight following stressed gestations. However, an opposing fetal response still indicates higher male responsivity, potentially modified by maternal physiological and behavioral changes that drive increases in maternal glucose.

Increases in body size, particularly an increase in fetal fat reserves, could act adaptively in that they provide the infant with greater nutritional reserves when a stochastic external environment is signaled through maternal stress markers. This male responsivity indicates greater environmental sensitivity and potentially greater need for nutritional buffering due to larger average body size at birth. For example, one recent study finds that male fetal growth is accelerated under conditions of mild stress [69]. However, such an adaption would incur higher mortality risks during parturition, as well as potential negative downstream effects on adult health, lowering the likelihood that the response is adaptive.

However, it is equally likely that increase male macrosomia simply reflects the confluence of established male sensitivity and stress effects on maternal glucose. This response may further interact with the influence that fetal sex appears to have on maternal glucose regulation. Recent research shows that mothers of male fetuses have greater odds of developing GDM (odds ratio: 1.39, 95% CI: 1.01–1.90) than those carrying females. Carrying a male also further increases the effect size of usual GDM risk factors, such as older maternal age (>35 years) and non-White ethnicity [70].

Notably, the timing of the greatest effect differs in this work from that of several previous studies. Previous studies find that prenatal stress is most impactful in early pregnancy [4, 5, 34], although, when stress is nutritional rather than psychosocial more variety in the timing of effects has been observed [71]. Our work finds the greatest increases in birth weight amongst the cohort exposed in the second half of pregnancy, followed by those exposed to the fire aftermath in early pregnancy. Reduced responsivity to maternal cortisol in late pregnancy may explain the rarity of observable effects on birth weight in later pregnancy. However, because some responsivity remains, the effects of cortisol on blood glucose may still provide a physiological explanation for the relationship between late pregnancy stress and fetal weight found in this study.

## CONCLUSIONS AND IMPLICATIONS

We find a pattern of fetal macrosomia associated with maternal exposure to the 2003 Canberra wildfires among male neonates, which is contrary to other similar studies in that it finds a primary pattern of macrosomia accompanied by small increases in lighter births, rather than the inverse pattern found elsewhere [36, 37].

The study has a number of limitations. First, the definition of disaster impact assumes both a relationship to geographical area and a causal link between disaster proximity and maternal stress. Although disaster impact is not necessarily geographically limited, no stronger method of defining disaster exposure is available when working within the constraints of public datasets and this approach is commonly used in the literature. Second, we are limited in the specificity of our estimates of the timing of fetal exposure to the fire, as birth dates were only identified by month and year, limiting the specificity of that measure. Lastly, we are unable to directly test our hypothesis regarding maternal blood glucose elevation. This is an area which we hope to examine more closely in future work.

Nevertheless, the findings of the study indicate a risk of macrosomia to exposed male infants. Fetal macrosomia poses immediate health risks to mother and child, as well as longer term health risks to the child. Our analysis finds that neither changes to maternal cigarette smoking nor changes to diagnosed rates of GDM in the population are good explanations for the observed pattern. However, elevations in maternal glucose can be sufficient to increase fetal weight without being sufficient to trigger a GDM diagnosis. Therefore, we propose that the maternal stress response precipitated an increase in maternal glucose levels that accelerated fetal growth in the more environmentally responsive male cohort. Disaster-related behavioral changes, such as a higher calorie diet and reduced physical activity, might have exacerbated this process.

While our finding is contrary to the general trend of prematurity and lowered birth weights associated with disaster stress, this contrary finding does not necessarily imply that the underlying evolutionary features of the changes are similarly altered. Our findings accord with both theories of increased male frailty, as demonstrated through greater environmental responsivity, and that maternal stress acts as an indicator of future environmental quality. Potentially, higher birth weight is simply a consequence of maternal glucose rising in response to stress; however, it is also plausible that increased body weight is an adaptive response to expected future stress, albeit compromised by heightened immediate survival risks and downstream health risks.

Although the timing of effects found here differs from previous work, we propose an underlying evolutionary explanation for the observed pattern. In late pregnancy, maternal investment is high and the risk of fetal loss low, because of the sunk costs of maternal investment thus far. Due to this high investment, mothers experiencing late pregnancy stress may opt for a ‘double or nothing’ strategy in which maternal investment increases to boost the chance of neonatal survival and consequent return on investment. While this strategy may disadvantage a small number of larger neonates by increasing their risk of macrosomia and its complications, for the majority such a strategy would provide an unproblematic increase in body weight which provides a buffer against environmental stress.

Importantly, our results are consistent with previous findings in that they find an association between disaster exposure and changes in birth outcomes that threaten maternal and fetal health. Thus, the broader implementation of maternal stress management as an aspect of disaster response is lent further support by this work.
